# Characterization of fecal sludge as biomass feedstock for thermal treatment in the southern Indian state of Tamil Nadu

**DOI:** 10.12688/gatesopenres.12870.2

**Published:** 2020-01-24

**Authors:** Viswa Barani, Meghan Hegarty-Craver, Praveen Rosario, Prakash Madhavan, Prasanna Perumal, Sarani Sasidaran, Milan Basil, Antony Raj, Adrian B. Berg, Andrea Stowell, Camille Heaton, Sonia Grego

**Affiliations:** 1 PSG Institute for Medical Sciences and Research, Coimbatore, TN, 641004, India; 2 RTI International, Research Triangle Park, NC, 27709, USA; 3 RTI International - India, New Delhi, 100037, India; 4 Center for WaSH-AID, Duke University, Durham, NC, 27701, USA

**Keywords:** total solids, higher heating value, ash content, heavy metal, proximate analysis, ultimate analysis

## Abstract

**Background**: Transformative sanitation technologies aim to treat fecal sludge (FS) by thermal processes and recover resources from it. There is a paucity of data describing the relevant properties of FS as viable feedstock for thermal treatment in major geographical target areas, such as India.

**Methods**: This study characterized FS collected from septic tanks in two cities located in the Indian southern state of Tamil Nadu. FS samples were obtained at the point of discharge from trucks in Tiruppur (n=85 samples) and Coimbatore (n=50 samples). Additionally, biosolids obtained from sewage treatment plants (STP) in the cities of Coimbatore and Madurai were characterized. Total solids (TS) were measured, and proximate and ultimate analysis were conducted according to methods used by the fuel industry. Additionally, the ash content was analyzed for heavy metal using standard methods. **Results: **The average higher heating value (HHV) across all FS samples in Tiruppur (13.4 MJ/kg) was significantly higher than in Coimbatore (5.4 MJ/kg), which was partially attributed to the high ash content of 69% in the latter samples.  The HHV of the biosolids samples ranged from 10 to 12.2 MJ/Kg. The average total solids (TS) content for FS was 3.3% and 2.0% for Tiruppur and Coimbatore respectively, while the median TS content for the two cities was 2.3% and 1.2%. The heavy metal content of the ash was found to be below the thresholds for land disposal.

**Conclusions: **This is one of the first studies that has systematically characterized the calorific and mineral content of septage and biosolids in several cities in India. We expect these data to serve as input data in the design of thermal processes for fecal sludge treatment.

## Introduction

Access to safe water and proper management of human waste is a major global challenge and is one of the United Nations Sustainable Development Goals. As much as a third of the world population (2.4 billion people) lacks access to improved sanitation ( WHO/UNICEF, 2014). Of these, 800 million live in India, and 650 million live in sub-Saharan Africa ( WHO/UNICEF, 2014).

Developing and emerging countries rely extensively on onsite waste treatment (e.g., septic tanks or pit latrines). In India, only 32.7% of the urban population has a piped sewer connection ( Census of India, 2011). Fecal sludge (FS), which is the raw or partially digested semisolid material that is produced from human excreta and blackwater, collects in onsite technologies and needs to be removed periodically. The economic burden of providing adequate treatment of fecal sludge results in unsafe discharge of waste emptied from onsite systems and potential health consequences.

Resource recovery from waste may support the development of viable business models for sustainable sanitation solutions ( Diener *et al*., 2014). Traditionally, the most common form of resource recovery from fecal sludge solids has been that of soil conditioning. More promising options have recently emerged including use of fecal sludge as components in building materials, as a source of protein for animal feed and as industrial fuel ( Diener *et al*., 2014). 

Innovative sanitation treatment approaches under development aim to recover energy from onsite waste systems through processes including combustion ( Sellgren *et al*., 2017), gasification ( Onabanjo *et al*., 2016), smoldering ( Yermán *et al*., 2015), hydrothermal oxidation ( Miller *et al*., 2015) and hydrothermal carbonization ( Afolabi *et al*., 2017). Key figures of merit for biomass feedstock include total solids (TS) content and calorific content. FS characterized by a low TS and/or calorific content poses significant technical challenges for energy recovery applications, making these critical parameters for determining the economic viability of the use of FS as fuel.

The calorific content, expressed as higher heating value (HHV), of fresh human feces is approximately 21 MJ/kg, which compares well to wood biomass ( Onabanjo *et al*., 2016). The energetic properties of sludge depends heavily on the type of treatment used ( Fonts *et al*., 2009). Values ranging from 25 MJ/Kg for primary sewage sludge treated at municipal waste treatment facilities ( Tyagi & Lo, 2013) to 9–12 MJ/kg for anaerobically digested sewage sludge ( Fonts *et al*., 2009) have been reported. 

There is a lack of information related to the calorific properties of septage, or fecal sludge exclusively from septic tanks, in developing countries. For onsite sanitation systems, a study from three cities in west Africa reported HHV between 16–19 MJ/kg (for dried solids), and TS content ranging from 1% for septic tanks to 6% for unlined pit latrines ( Muspratt *et al*., 2014). A recent report from Ghana also found TS values of 1–2% in septic tanks and 5% in pit latrines ( Fanyin-Martin *et al*., 2017).

Septage properties are expected to be system- and location-dependent due to different structural and environmental conditions and personal hygiene habits, particularly whether toilet paper is used or, as in India, water is used for cleansing. Recently, a survey of the septage composition in the metropolitan area of Chennai, India reported very low solids content (0.2–0.35%) ( Krithika *et al*., 2017).

The southern state of Tamil Nadu, one of the most industrialized and populous states in India, provides a suitable environment for new technologies for FS management. This group of investigators and other teams funded by the Bill & Melinda Gates Reinvent the Toilet initiative have been conducting field studies related to technologies for FS thermal processing in this region since 2015.

The present study addresses the knowledge gap related to the physical and energetic properties of FS as a potential fuel source in southern India. Fecal sludge from septic tanks was sampled from desludging trucks in the urban areas of Coimbatore and Tiruppur in the southern state of Tamil Nadu. The biosolids (i.e., treated sewage sludge) generated at sewage treatment plants (STP) represents another potential biomass feedstock for thermal processing and was also collected. The TS and calorific content (HHV) were measured and proximate and ultimate analysis were conducted according to standard procedures used in the fuel industry. The ash content was also characterized for heavy metals that pose a potential environmental hazard.

## Methods

### Site descriptions

We investigated the properties of the FS and biosolids in three cities located within a 200km radius in the southern state of Tamil Nadu: Coimbatore, Tiruppur, and Madurai. Coimbatore has a population of 1.6 million, and the average temperature and rainfall are 26.3°C and 618mm. Tiruppur has a population of 0.88 million, and the average temperature and rainfall are 27.3°C and 605mm. Madurai has a population of 3 million, and the average temperature and rainfall are 28.8°C and 840mm. Regionally, precipitation is lowest in January-February (7–14mm), and highest in October (151–191mm) (See climate-data.org). A description of the sample collection and details of the STPs in the three cities is provided in Table 1. Table 1 also includes the city sewage coverage as estimated by respective city officials (personal communication).

**Table 1.** Breakdown of sample collection by city. City estimated sewage coverage and details of the sewage treatment plants (STP) are included. MLD (Million-liter per day).

	Coimbatore	Tiruppur	Madurai
**Sewage coverage**	~ 7%	~17–18%	~70%
**Fecal Sludge (FS) sampling**	Yes	Yes	No
**Biosolids sampling**	Yes	No	Yes
**Sewage Treatment Plant (STP)**	Ukkadam	Not evaluated	Avaniyapuram (AV) and Sakki Magalam (SK)
**Flow to STP (MLD)**	25–30	-	20–25/10–15
**Collection date**	November 2016, Jan 2017	Jan 2017, July 2017, Feb-March 2018	December 2016

The properties of both the fecal sludge delivered to the Coimbatore Ukkadam STP, as well as the biosolids generated at the Ukkadam STP, were analyzed.

Sample collection efforts in Tiruppur were coordinated through contact with multiple septic tank service companies, and meetings were arranged at different discharge sites (i.e., fields or open pits) located throughout the city. Biosolids samples were not available in this city. 

Two STPs were identified in Madurai (Avaniyapuram and Sakki Magalam) and the biosolids generated by each STP were analyzed. FS samples were not studied in Madurai.

### Fecal sludge sampling

Due to logistical constraints, sample collection from desludging trucks in Coimbatore occurred on two separate days in November 2016 and January 2017, both in the dry season. Fifty samples were collected by randomly selecting desludging trucks that were discharging their load at the Ukkadam STP. Multiple sampling campaigns were undertaken in Tiruppur across a 14-month period (Jan 2017, July 2017, Feb–March 2018) that included both the dry and wet seasons. In total 85 samples were collected at this location.

Information related to the source of the sample was collected and included: location, location category (i.e., residential, public, commercial, and industrial), additional category details (public community toilet, house, apartment, etc.), type of sanitation system (septic tank and soak pit), inclusion of graywater (yes/no), and maintenance quality of system (poor, moderate, and high).

The truck volumes ranged from 4000–7500 liters in Coimbatore, and 3500–8000 liters in Tiruppur. A stopwatch was used to measure the amount of time that it took the truck to discharge (the typical range was 3–7 minutes). Five liter containers were used to collect grab samples, which were taken every 30 s beginning with initial discharge. The grab samples from the three containers representing the beginning, middle, and end of emptying, were combined to make a 15L composite sample for each truck. After thoroughly mixing with a clear rod, a 4.5L sample volume was collected for further processing and analysis, and the remainder was discarded.

The 4.5L composite FS samples from each truck were individually solar dried in large plastic bins that were partially covered with plastic sheets. The bins were dried over the course of multiple days and stored indoors overnight. This solar drying reduced the sample volume to a wet sludge that was transferred to petri dishes for oven drying. The petri dishes were placed overnight in a temperature-controlled oven that did not exceed 100°C (typically 55°C) in order to ensure that there was no biomass loss due to volatilization during the drying process. This temperature differs from the standard value of 105°C because the oven available for this project did not control the temperature with adequate precision. After overnight drying, the samples were removed from the oven and allowed to cool for 15 minutes, and the dry mass measured by a laboratory scale. The samples were placed back in the oven for an additional 4 hours, and the mass re-measured. This process was repeated until the dry mass varied by <4%, according to APHA 2540 B. The samples were then lightly ground and mixed by hand in a plastic tub to form a homogenous composite mixture.

### Biosolids sampling

Biosolid samples were obtained from fecal sludge that was processed at the Ukkadam STP in Coimbatore, and the Avaniyapuram and Sakki Magalam STPs in Madurai. Due to logistical constraints, biosolids samples were collected on two separate days in December 2016 in Madurai and January 2017 in Coimbatore; sample collection in both cities occurred during the dry season.

The biosolids samples were collected soon after being processed according to the plant standard procedure. The processing apparatus was located on the first floor of each of STPs, and a trailer was parked underneath to collect the wet biosolids. During normal operation, the trailer is used to transport the wet biosolids to an adjacent drying field.

For sample collection, the trailer bed was thoroughly rinsed to remove potential contamination. A rope and tape measure were used to demarcate six equal segments of the bed of the trailer. A custom trier sampler was pressed straight down into the center of each section, and then carefully extracted. Excess biosolids were cleaned from the outside of the trier sampler, and its contents added to a 20 liter mixing bucket. Sampling was conducted in this way for the remaining five sections, and the composite sample was thoroughly mixed. Then, the samples were transferred to the transport containers and sealed.

The biosolids samples were dried in petri dishes to measure total solids using the same procedure described above for the FS samples. 

### Sample analysis

The dried solids content from the individual trucks in Coimbatore and Tiruppur were combined by date or by category prior to proximate and ultimate analysis.

Duplicate 500 gram samples of the dried FS or biosolids were shipped in sealed containers to SGS India Pvt. Ltd (Chennai laboratories) for analysis.

HHV was measured by bomb calorimetry according to ASTM D5865. For the proximate analysis tests, the ash content was determined according to ASTM D3174, volatile matter was determined according to ASTM D3175, the percent sulfur was determined using the bomb washing method according to ASTM E 775, and fixed carbon was determined according to ASTM D3172. 

For the ultimate analysis tests, the percent carbon, hydrogen and nitrogen were obtained according to ASTM D5373, total sulfur content was determined according ASTM 4239 (high temperature combustion with thermal conductivity detection), and the percent oxygen obtained according ASTM D3176 by difference of CHNS and ash content. 

Ash composition analysis was carried out according to ASTM D6349 by ICP-MS/ICP-OES (inductively coupled plasma optical emission spectrometry) for oxides and mercury, and trace metals were measured with in-house standard operating procedure for ICP.

### Empirical models

To ensure consistency between bomb calorimetry and elemental analysis, estimates of the HHV were calculated from the elemental content reported in the proximate and ultimate analysis using two alternative formulas from the literature:

Equation 1 ( Channiwala & Parikh, 2002)

*HHV(MJ/Kg) =0.3491 C+1.1783 H+0.1005 S-0.1034 Y-0.0151 N-0.0211 A [1]*

Equation 2 ( Sheng & Azevedo, 2005)

*HHV (MJ/Kg) =-1.3675+0.3137 C+0.7009 H+0.0318 Y                             [2]*

where C = % carbon, H = % hydrogen, S = % sulfur, Y = % oxygen, N = % nitrogen, A = % ashweight percent on a dry mass basis.

The lower heating value LHV in MJ/Kg was calculated by HHV using the relationship from ( IPCC, 2006)

LHV (MJ/Kg) =HHV(MJ/Kg) -0.212*H- 0.008*Y                                      [3]

### Statistical analysis

Data analysis was conducted using Microsoft Excel 2016. The TS content was measured for each sample on an individual basis, and statistical analysis performed for all samples from each location separately. Proximate and ultimate analysis, as well as ash content, were characterized for at least two replicates, and statistical analysis performed by location. The results reported below represent averaged values over the number of specimens (indicated by n), and error bars are used to indicate the standard deviation.

## Results and discussion

### Description of FS sources

Samples were collected from n=50 truck discharges serving distinct locations in Coimbatore on two separate days, and n=85 truck serving distinct locations within Tiruppur over multiple sampling days. Based on the information collected by surveying the drivers, most of the septic systems were reported to have good (high or moderate) maintenance, with only a few (3/85 for Tiruppur and 3/50 for Coimbatore) reporting poor or unknown maintenance of the onsite system. Septic tanks accounted for 90% or more of the sampled systems, with soak pits accounting for 10% of the samples in Coimbatore and 4% of the samples in Tiruppur.

Based on the survey information, the FS sources were assigned to one of five categories:

1. residential single-family houses
2. residential multifamily establishments (e.g. apartments, or community houses with one common septic tank)
3. industrial sites
4. commercial sites
5. public toilets

**Figure 1.**
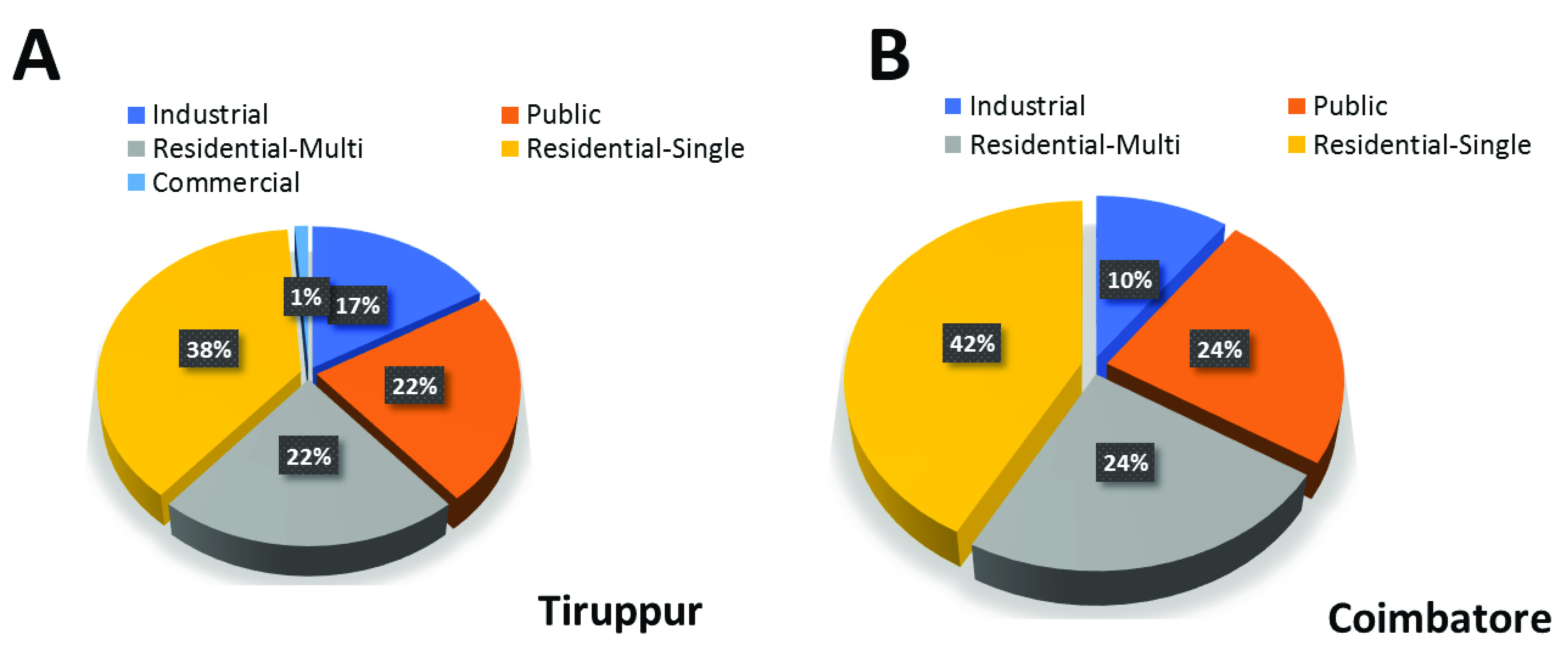
Characterization of fecal sludge sample sources **A**. Tiruppur **B**. Coimbatore.

Figure 1 illustrates the distribution across categories of the sampled sources for Tiruppur and Coimbatore. The distribution across the five categories is similar between the two cities. The majority of the collected samples were from residential sites accounting for both single and multi-family units (60% and 66% of the total number for Tiruppur and Coimbatore, respectively), with the second major grouping coming from public toilets (22% and 24% of the total number for Tiruppur and Coimbatore, respectively).

### Total solids content of FS sources

Table 2 summarizes descriptive statistics on the TS values. The average TS in Tiruppur (3.3%) is higher than in Coimbatore (2%), and also higher than the 1–2% reported in the literature for septic tanks in Africa ( Fanyin-Martin *et al*., 2017; Muspratt *et al*., 2014). The TS values measured in Coimbatore and Tiruppur are an order of magnitude higher than the 0.2–0.3% reported for the metropolitan area of Chennai, which is also located in the state of Tamil Nadu ( Krithika *et al*., 2017). However, based on the driver survey results, only 36% of the sources in Coimbatore and 11% of the sources in Tiruppur received graywater in addition to blackwater. In India, it is common for only the toilet effluent to be plumbed to the septic tank while graywater is discharged to an open drain ( Ministry of Urban Development, 2011).

**Table 2.** Descriptive statistics of the total solids contents measurement of fecal sludge.

Parameter	Tiruppur (n=85)	Coimbatore (n=49)
**Average**	3.3 %	2.0 %
**Standard Deviation**	2.8 %	2.3 %
**Median**	2.3 %	1.2 %
**Min**	0.1 %	0.02%
**Max**	16.8 %	9.9 %

While the percentage of sources including graywater relies on self-reported data, this data aligns well with the higher values of TS measured in settings with low percentage of graywater inclusion, and is comparable to septage in African cities ( Fanyin-Martin *et al*., 2017; Muspratt *et al*., 2014). The study by Krithika and colleagues that took place in a geographically similar region did not track graywater inclusion, but did report a very high emptying frequency (from days to one month), which suggests the likelihood of graywater being included ( Krithika *et al*., 2017).

**Figure 2.**
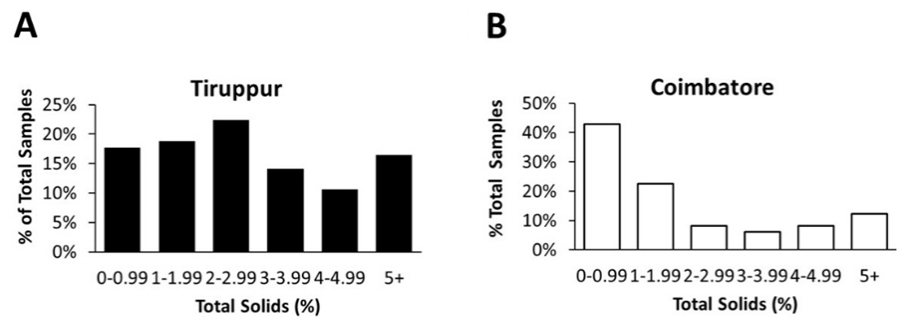
Distribution of total solids for fecal sludge samples by city.

The range of TS values observed in Coimbatore and Tiruppur is large and broadly distributed ( Figure 2). The average TS is skewed high due to a few samples with a very high TS concentration (larger than 5%), so the median value is a more representative estimate of the FS volume available as biomass feedstock in these cities. The shape of the TS distribution is different between the two cities, with Coimbatore having a large fraction of the samples below 1% while samples from Tiruppur are more uniformly distributed between 1–5 %. This study did not examine the effect of different seasons on the TS content, which has been reported to decrease during the rainy seasons in Tamil Nadu ( Krithika *et al*., 2017). For the city of Tiruppur, sampling was carried out during both seasons, but no significant difference was found.

### Effect of multiple desludging trips

**Figure 3.**
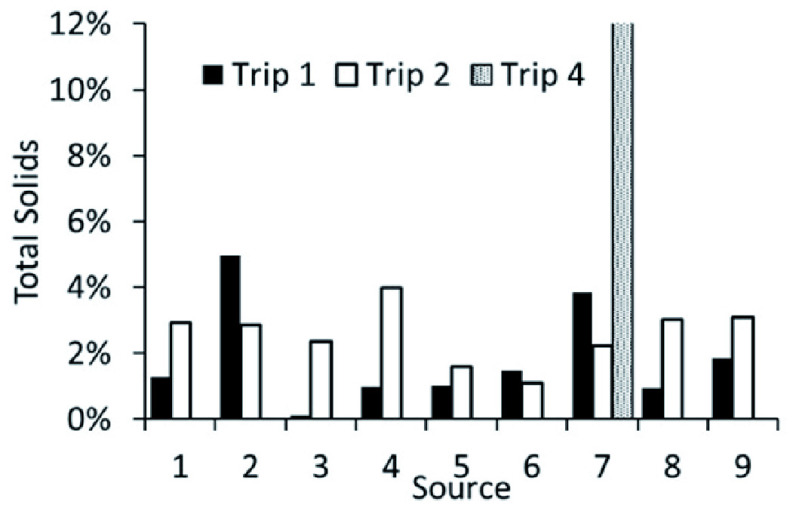
Multi-trip total solids (TS) results from Tiruppur. Source 7 required four consecutive trips, all other sources required two trips.

This study was able to evaluate samples drawn from trucks conducting multiple trips to fully empty the same septic tank. Figure 3 reports the TS measured in successive trips for nine sources. We anticipated that the TS content from the first trip would be lower because this sample would represent the upper liquid layer of the tank. In 6 out of 9 measurements, the TS concentration from the second trip was indeed higher than the first. Source site 7 was a particularly large septic tank and required four trips to be emptied (samples were collected from trips 1, 2, and 4). The TS concentration from the fourth and final trip was extremely high, indicating that this sample likely represented the dense sludge at the bottom of the septic tank.

### Elemental composition and calorific content of FS sources

For Coimbatore, 29 trucks were combined into one composite, and two replicates analyzed (N=2). For Tiruppur, samples from different source categories were combined (pilot n=12, public n=20, industrial n=13, multi-family n=18, single-family n=21), and replicates of each (triplicate for public and duplicate for all other categories) were analyzed for a total of N=11 samples. A higher number of specimens were analyzed in Tiruppur than in Coimbatore for pragmatic reasons related to an opportunity of piloting novel decentralized FS thermal treatment in that city.

The results of the proximate and ultimate analysis by city are provided in Table 3.

**Table 3.** Proximate and ultimate analysis of the moisture-free fecal sludge samples (Avg +/- St. Dev). N is the number of replicates analyzed. HHV (higher heating value), LHV (lower heating value).

	Coimbatore	Tiruppur
**Proximate Analysis**	**N = 2**	**N = 11**
**Ash (%)**	69.3 ± 12.9	39.0 ± 12.8
**Volatile Matter (%)**	26.5 ± 8.8	47.7 ± 9.9
**Fixed Carbon (%)**	3.2 ± 3.5	11.4 ± 3.2
**HHV [Meas] (MJ/kg)**	5.4 ± 2.4	13.4 ± 3.2
**Sulfur (%)**	0.8 ± 0.2	1.1 ± 0.2
**Ultimate Analysis**	**N = 2**	**N = 11**
**Carbon as C (%)**	16.3 ± 6.7	33.0 ± 7.4
**Hydrogen as H (%)**	2.4 ± 1.2	4.7 ± 1.0
**Nitrogen as N (%)**	1.4 ± 0.7	3.1 ± 0.7
**Oxygen as O (%)**	9.7 ± 4.1	19.2 ± 4.1
**Sulfur as S (%)**	0.9 ± 0.2	1.1 ± 0.2
**HHV [Equ 1] (MJ/kg)**	6.1 ± 3.6	14.3 ± 3.7
**HHV [Equ 2] (MJ/kg)**	5.8 ± 3.1	13.0 ± 3.1
**LHV [Equ 3] (MJ/kg)**	5.2 ± 3.8	11.9 ± 2.9

The average ash content in Coimbatore (69%, N=2) was much higher than in Tiruppur (39%, N=11), the latter being similar to ash values reported in the literature for sewage ( Fonts *et al*., 2009). Because the ash content of fresh feces is only 17% (on a dry basis) ( Onabanjo *et al*., 2016), the higher ash content of sludge could be attributed to environmental factors such as the construction practices of onsite systems or to a longer storage time leading to increased degradation. A high ash content is detrimental to the calorific value of the sludge. The HHV of the sludge sampled in Coimbatore was low (5.4 MJ/kg), while the sludge sampled from Tiruppur had a higher HHV (13.4 MJ/kg) with a higher fixed carbon level. Elemental analysis revealed higher percentages of carbon and oxygen in Tiruppur compared to Coimbatore, which is in agreement with higher HHV (see Equation 1 and Equation 2).

**Figure 4.**
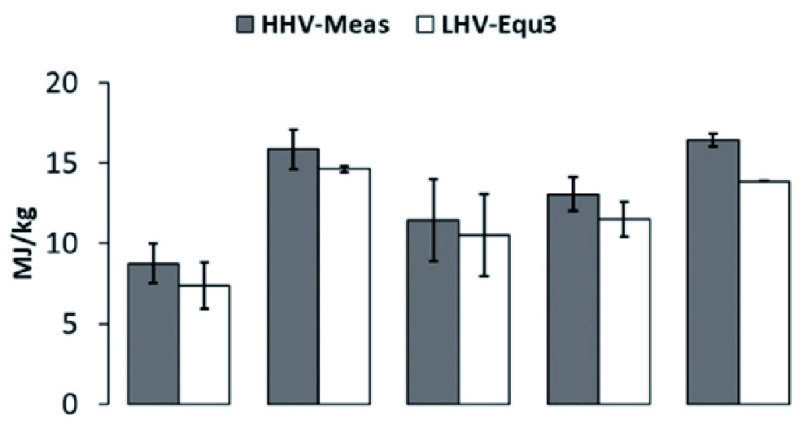
Calorific content of the fecal sludge in Tiruppur. Measured HHV and LHV calculated according to Equation 3 by source category: pilot study, public community toilet, industrial, multifamily, and single family establishment (errorbar: st. dev).

The analysis for the dried composite samples for Tiruppur further enabled segmentation of the HHV by sludge source ( Figure 4). The pilot sites featured the lowest HHV values measured for this city for undetermined reasons. The industrial sites featured lower HHV than residential sites, particularly the single-family sites. The FS from public toilets also exhibited a high HHV. The LHV, calculated from measured HHV using Equation 3, is reported in Figure 4 to illustrate the calorific value available for thermal processes that do not capture the latent heat of water vaporization and it is therefore a more conservative measure of the calorific value of the biomass feedstock compared to HHV.

### Heavy metal content of FS sources

Thermal processing of fecal sludge produces ash streams (either bottom ash or fly-ashes) that ultimately need to be disposed of. The heavy metals in sludge or biomass become concentrated into ash upon thermal processing ( Nzihou & Stanmore, 2013) creating a concern for disposal of ash, which may be labelled as hazardous waste if values exceed certain thresholds. We measured the concentration of the following metals in the FS ash: barium (Ba), selenium (Se), silver (Ag), arsenic (As), cadmium (Cd), chromium (Cr), lead (Pb) and mercury (Hg).

Table 4 reports the average of the two samples measured for Coimbatore, as well as each sludge source category in Tiruppur. A third replicate for the Tiruppur public community toilet sample was removed as an outlier (these results are included in the supplemental data provided with this report). The heavy metal results were compared with values from literature, as well as thresholds defined by the United States Environmental Protection Agency (EPA) for land disposal and by India Ministry of Environment, Forest and Climate change (MOEF) for organic compost.

**Table 4.** Heavy metal content of fecal sludge ash samples (samples n=2 for each category) in mg/kg dry mass. Average ± st. dev. is reported.

Parameter (mg/kg)	Coimbatore	Tiruppur Pilot	Tiruppur Community	Tiruppur Industrial	Tiruppur-Multi	Tiruppur Single
Ba	108.5 ± 9.2	191.0 ± 17.0	>1000	140.5 ± 4.9	150.5 ± 10.6	160.0 ± 7.1
Se	<10	<10	<10	<10	<10	<10
Ag	<10	<10	<10	<10	<10	<10
As	<10	<10	<10	<10	<10	<10
Cd	<10	<10	<10	<10	<10	<10
Cr	22.0 ± 9.9	60.0 ± 70.7	79.0 ± 9.9	<10	<10	<10
Pb	10.0 ± 1.4	10.5 ± 0.7	21.0 ± 2.8	<10	<10	<10
Hg	<10	<10	<0.5	<0.5	<0.5	<0.5

We found that selenium, silver, arsenic, and cadmium were below the measurement detection limit of 10mg/kg in both cities and all categories. Barium content was found to be high (>100mg/kg) in all the measurements. The barium values are similar to values reported from the FS study in Chennai India ( Krithika *et al*., 2017), as well as to values measured in a study of biomass combustion in China ( Li *et al*., 2012). Although Barium does not have a threshold concentration in solid waste, there is an EPA limit of 100 mg/L as a leachate for land application.

Chromium concentrations of 20–80 mg/kg were detected in Coimbatore, as well as two different sample sets from Tiruppur; the concentration was below 10 mg/kg for the other samples. These values are comparable or smaller than values found in the literature ( Krithika *et al*., 2017; Li *et al*., 2012), and lower than the US EPA pollutant concentration limit of 200 mg/Kg for disposal. The MOEF threshold for organic compost is 50mg/kg for chromium, which was not achieved for several of the surveyed samples.

Lead values were found to be low, between 10–20 mg/kg (at most), meeting both the EPA pollutant concentration limit of 300 mg/kg for disposal and MOEF limit of 100 mg/kg for compost.

Mercury was measured below the detection limit in all samples. Mercury levels are therefore below the EPA ceiling concentration for disposal of 57 mg/kg, however, the measurement detection limit was not adequate to establish whether the MOEF threshold of 0.15mg/kg was met.

Comparing the sludge source categories with each other ( Table 4), the community toilet samples have the highest heavy metal content. We speculate that they may be more prone to disposal of polluting waste because they are the least controlled environment of all tested sources.

### Properties of biosolids sources

Biosolids were analyzed from Ukkadam STP in Coimbatore, and the Avaniyapuram (AV) and Sakki Magalam (SK) STPs in Madurai. Table 5 summarizes the measured values for TS, as well as the results of the proximate and ultimate analysis. The TS content of the biosolids is much higher than the FS and has a narrower distribution of values (range: 16.5%–18.9%). Ash content was also very similar across sites. HHV values ranged between 10 and 12 MJ/kg, with the Sakki Magalam samples exhibiting the highest fraction of carbon and highest HHV. The HHV of the Coimbatore biosolids was 11 MJ/Kg, which is much higher than the values measured for septage from the same city (5.4 MJ/kg). The LHV was calculated to be between 9–11 MJ/kg.

**Table 5.** Proximate and ultimate analysis of biosolid samples on a dry basis. Average +/- st dev. HHV (higher heating value), LHV (lower heating value), Madurai AV (Avaniyapuram) and SK (Sakki Magalam).

	Madurai-AV	Madurai-SK	Coimbatore
**Samples**	**N = 4**	**N = 4**	**N = 9**
**Total Solids (%)**	18.2 ± 1.6	16.5 ± 0.7	18.9 ± 2.4
**Proximate Analysis**	**N = 4**	**N = 3**	**N = 4**
** Ash (%)**	48.3 ± 0.1	47.4 ± 9.3	47.9 ± 4.3
**Volatile Matter (%)**	46. 6 ± 2.8	47.5 ± 5.8	45.7 ± 0.8
**Fixed Carbon (%)**	4.1 ± 3.1	3.7 ± 3.5	4.0 ± 4.5
**HHV [Meas] (MJ/kg)**	10.0 ± 0.5	12.2 ± 1.6	11.0 ± 0.6
**Sulfur (%)**	1.4 ± 0.2	1.3 ± 0.1	1.4 ± 0.1
**Ultimate Analysis**	**N = 4**	**N = 3**	**N = 4**
**Carbon as C (%)**	26.2 ± 0.1	33.4 ± 0.6	29.0 ± 1.3
**Hydrogen as H (%)**	3.5 ± 0.1	4.2 ± 0.3	3.6 ± 0.3
**Nitrogen as N (%)**	3.5 ± 0.0	4.5 ± 0.2	2.6 ± 0.1
**Oxygen as O (%)**	17.0 ± 0.1	9.3 ± 8.2	15.5 ± 3.0
**Sulfur as S (%)**	1.4 ± 0.2	1.3 ± 0.1	1.5 ± 0.1
**HHV [Eq 1] (MJ/kg)**	10.5 ± 0.2	14.7 ± 0.1	11.9 ± 0.6
**HHV [Eq 2] (MJ/kg)**	10.0 ± 0.1	12.5 ± 0.6	10.9 ± 0.7
**LHV [Equ 3] (MJ/kg)**	9.1 ± 0.5	11.2 ± 1.5	10.1 ± 0.6

### Empirical models

**Figure 5.**
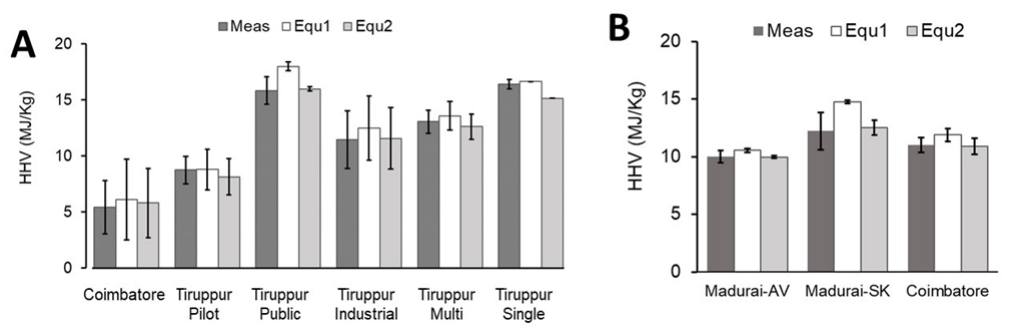
Higher heating value (HHV) by city and source category measured and calculated according to Equation 1 and Equation 2, by city and source category. **A**. Fecal sludge (FS) samples and **B**. biosolids samples. Error bar is the st. dev.

The elemental composition of both the FS and biosolids samples was used to estimate HHV according to two distinct empirical models reported in the literature. The first model (see Equation 1) is commonly used ( Channiwala & Parikh, 2002), and the second model (see Equation 2) represents a more recent approach ( Sheng & Azevedo, 2005). Figure 5 reports the comparison of the measured HHV values and the calculated values according to the two models for the five categories of FS and three biosolids sites. We found that the measured HHV compared well with the empirical model results, with the model developed by Sheng *et al*. consistently being the more accurate predictor of the measured HHV (the average absolute error across our dataset for the Sheng model is 0.38 MJ/kg vs. 0.95 MG/kg for the Channivala model).

### Applicability of thermal processes to fecal sludge treatment

Thermal processing of human waste is an attractive sanitation solution because it provides safe sanitation treatment while offering the opportunity of resource recovery in the form of fuel gases and solid nutrients. Thermal processes for FS treatment and resources conversion are significantly impacted by the moisture content of the feedstock and the selection of a suitable process must fit the properties of the waste stream.

The data obtained from the biosolids in Coimbatore and Madurai indicates that the calorific values (HHV ~ 9–11 MJ/kg) are degraded compared to those of fresh feces (HHV ~ 21 MJ/kg) and that that the solid content (16–19%) is reasonably high, thereby biosolids provide a feedstock suitable for many types of thermal processes to be centralized at the sewage treatment plant.

Smoldering ( Yermán *et al*., 2015), gasification ( Onabanjo *et al*., 2016), and pyrolysis ( Yacob *et al*., 2018) have been evaluated for the decentralized treatment of human feces, which typically has a moisture content of ~77%. Decentralized treatment is advantageous to minimize the costs and risks of transporting waste to a treatment site. The average moisture content of the septage characterized in this study exceeded 97%. Thermal processes designed to handle high moisture content such as hydrothermal oxidation ( Miller *et al*., 2015; Qian *et al*., 2016) or hydrothermal carbonization ( Afolabi *et al.*, 2017) may be better suited for the treatment of this septage. Challenges associated with hydrothermal processes include scaling down to compact decentralized systems and the need for pre-thickening the feedstock to 5–10% solids ( Tyagi & Lo, 2013). Also the calorific value of FS was found to be degraded compared to fresh feces and varied significantly by location. 

Given the water stress currently experienced by India and in the state of Tamil Nadu in particular, innovative technical solutions addressing water conservation for toilet operation, as well as the development of compact and efficient dewatering technologies, may be helpful in reducing the moisture content of FS. Other systemic interventions such as improving construction practices and desludging frequency may be required to increase the calorific content of the feedstock for optimal resource recovery by thermal processing.

## Conclusion

This study presents an extensive characterization of the fecal sludge and biosolids from cities in the southern Indian state of Tamil Nadu, including HHV (i.e., calorific value), which is a critical figure of merit for biomass to be used as fuel. The HHV values for FS measured in two cities were quite different despite their geographical proximity. The HHV values measured for FS in Tiruppur, as well as for biosolids in both Coimbatore and Madurai, are at or above 10 MJ/Kg while the Coimbatore FS is much lower at 5.4 MJ/Kg. The heavy metal content of the FS ash in both cities indicates that it would not be considered as hazardous waste and, with some caveats, may be suitable for agriculture. The study evaluated FS from both residential and public settings and did not identify any major trend dependent on the generator.

In agreement with previous work, high variability in TS values from individual septic tanks was found. The averages TS value for the FS in septic tanks from two medium-sized cities in India were comparable with data from African studies (in the 1–3% range), and much higher than the values reported for a metropolitan area in same Indian state.

We expect this analysis to inform the design of thermal processes and systems to properly manage septage.

## Data availability

The data underlying this study is available from Open Science Framework (OSF), Dataset 1: “Fecal Sludge and Biosolids Sample Analysis (Tamil Nadu)”, https://doi.org/10.17605/OSF.IO/MDAUN ( Hegarty-Craver, 2018).

Raw data available

- File 1: Coimbatore Fecal Sludge Sample Information
- File 2: Tiruppur Fecal Sludge Sample Information
- File 3: SGS Fecal Sludge Results
- File 4: SGS Biosolids Results

Data are available under the terms of the Creative Commons Zero “No rights reserved” data waive (CC0 1.0 Public domain dedication).
