# Supplementary material for: Characterization of fecal sludge as biomass feedstock for thermal treatment in the southern Indian state of Tamil Nadu
Source: Gates Open Res. 2020 Jan 24;2:52. Originally published 2018 Oct 10. [Version 2] doi: 10.12688/gatesopenres.12870.2 (PMC7383100; doi:10.12688/gatesopenres.12870.2)
Supplement: Peer review history

# Peer review: Reviewer response for version 2

**DOI:** 10.21956/gatesopenres.14273.r28501

**Published:** 2020-07-23

**Authors:** Ewan McAdam

**Affiliations:** 1 School of Water, Energy and Environment, Cranfield University, Cranfield, UK

The corrections have appropriately dealt with the comments made by both reviewers. Thanks to the authors for the response.

Is the work clearly and accurately presented and does it cite the current literature?

Yes

If applicable, is the statistical analysis and its interpretation appropriate?

Yes

Are all the source data underlying the results available to ensure full reproducibility?

Yes

Is the study design appropriate and is the work technically sound?

Yes

Are the conclusions drawn adequately supported by the results?

Yes

Are sufficient details of methods and analysis provided to allow replication by others?

Yes

Reviewer Expertise:

water treatment, wastewater treatment, gas treatment, energy, separation processes

I confirm that I have read this submission and believe that I have an appropriate level of expertise to confirm that it is of an acceptable scientific standard.

# Peer review: Reviewer response for version 2

**DOI:** 10.21956/gatesopenres.14273.r28500

**Published:** 2020-04-01

**Authors:** Santiago Septien Stringel

**Affiliations:** 1 Pollution Research Group, University of KwaZulu-Natal, Durban, South Africa

The paper has addressed satisfactory to my comments. The paper meets with high quality criteria for its publication.

Is the work clearly and accurately presented and does it cite the current literature?

Yes

If applicable, is the statistical analysis and its interpretation appropriate?

Partly

Are all the source data underlying the results available to ensure full reproducibility?

Yes

Is the study design appropriate and is the work technically sound?

Yes

Are the conclusions drawn adequately supported by the results?

Yes

Are sufficient details of methods and analysis provided to allow replication by others?

Yes

Reviewer Expertise:

Thermal process (drying, pyrolysis, gasification, combustion, biochar); faecal sludge characterization (rheology, stickiness, physiochemical properties)

I confirm that I have read this submission and believe that I have an appropriate level of expertise to confirm that it is of an acceptable scientific standard.

# Peer review: Reviewer response for version 1

**DOI:** 10.21956/gatesopenres.13956.r26706

**Published:** 2019-07-19

**Authors:** Ewan McAdam

**Affiliations:** 1 School of Water, Energy and Environment, Cranfield University, Cranfield, UK

The central thesis of this manuscript is to provide greater detail on the potential for faecal sludge to be managed via a thermal oxidative/reductive pathway. The data is of interest and specifically to those seeking to develop means of managing thermal pathways for the destruction of faecal sludge to support safe disposal, and could conceivably be used as a resource with which to understand technology transfer into the field.

 The manuscript is well written and provides useful data on a specific faecal sludge source; data from the field such as this, is hard to find and so presents an important base of data for those involved in this subject area.

 Where the manuscript could perhaps improve is in enriching the narrative around the specificity of the focus of the study but also, to more clearly set the boundary conditions to clarify the significance of the solids concentration and HHV values reported. To expand on this:

1. The title does not clearly articulate thermal processing, which may help draw in interested readers.
2. The faecal sludge source that is characterised is quite specific, and so being specific in the title and abstract may help prospective authors to identify the value of the manuscript more immediately.
3. Based on the solids concentration and HHV of the sludge, together with the spatial and sampling differences, it would be useful to have a short paragraph/subsection to help contextualise how this data might inform the type of thermal process that is most suitable for this source/type of sludge (e.g. this may infer sub-critical water oxidation as more favourable due to the lower solids concentration), what scales may be appropriate for intervention with thermal processes for this type/source of sludge and whether upstream technical interventions could transition this technical selection - e.g. shorter residence times before emptying, better construction techniques for storage, avoidance of flushwater etc. - all of which could challenge scale, mode and point of intervention, as well as determining the best available technology.

Is the work clearly and accurately presented and does it cite the current literature?

Yes

If applicable, is the statistical analysis and its interpretation appropriate?

Yes

Are all the source data underlying the results available to ensure full reproducibility?

Yes

Is the study design appropriate and is the work technically sound?

Yes

Are the conclusions drawn adequately supported by the results?

Yes

Are sufficient details of methods and analysis provided to allow replication by others?

Yes

Reviewer Expertise:

water treatment, wastewater treatment, gas treatment, energy, separation processes

I confirm that I have read this submission and believe that I have an appropriate level of expertise to confirm that it is of an acceptable scientific standard, however I have significant reservations, as outlined above.

# Response

**Published:** 2020-01-16

**Authors:** Sonia Grego

**Affiliations:** Duke University, USA

**We thank Dr. McAdam for the review and we appreciate his suggestions on how to better draw interested readers to the manuscript. Point by point response to his comments follow.**

 Where the manuscript could perhaps improve is in enriching the narrative around the specificity of the focus of the study but also, to more clearly set the boundary conditions to clarify the significance of the solids concentration and HHV values reported. To expand on this:

1. The title does not clearly articulate thermal processing, which may help draw in interested readers.
2. The faecal sludge source that is characterised is quite specific, and so being specific in the title and abstract may help prospective authors to identify the value of the manuscript more immediately

**In response to comments 1 and 2 the title and abstract have been modified to add specificity. In the title we added the word thermal treatment, but we were unable to be more specific on the type of FS because the paper reports on both septage and treated sewage sludge and we felt this is too much detail for a title. We specified FS types in the abstract.**

  

1. Based on the solids concentration and HHV of the sludge, together with the spatial and sampling differences, it would be useful to have a short paragraph/subsection to help contextualise how this data might inform the type of thermal process that is most suitable for this source/type of sludge (e.g. this may infer sub-critical water oxidation as more favourable due to the lower solids concentration), what scales may be appropriate for intervention with thermal processes for this type/source of sludge and whether upstream technical interventions could transition this technical selection - e.g. shorter residence times before emptying, better construction techniques for storage, avoidance of flushwater etc. - all of which could challenge scale, mode and point of intervention, as well as determining the best available technology.

 

**We embraced this comment and the insight shared by the reviewer and added a subsection entitled “Applicability of thermal processes to fecal sludge treatment” to discuss the implications of the data on the thermal processes that may best suit this environment.**

**In the subsection, we discuss separately the biosolids from the septage with emphasis on the opportunities for decentralized treatment, particularly by hydrothermal oxidation and added relevant references to the bibliography.**

# Peer review: Reviewer response for version 1

**DOI:** 10.21956/gatesopenres.13956.r26703

**Published:** 2018-10-22

**Authors:** Santiago Septien Stringel

**Affiliations:** 1 Pollution Research Group, University of KwaZulu-Natal, Durban, South Africa

This article is about the characterization of faecal sludge and biosolids from wastewater treatment plants from the province of Tamil Nadu, India, in view of reuse as biofuel. Available data about the faecal sludge characteristics in India is lacking in literature, whereas the implementation of efficient faecal sludge management is imperative in that country where there is a critical lack of sanitation. In this respect, this article presents useful data for sanitation practitioners for the implementation of faecal sludge treatment strategies in India, and builds in the body of knowledge about the characteristics of faecal sludge. In general, the article is well-written, structured and clear. The analysis of the results seems appropriate and the findings are consistent.

 I have no major comments on the article. Nevertheless, there are some minor points where the article could be improved and these are arisen in the list of comments below. Once the authors will address adequately these points, I will recommend the acceptance of the paper for its publication.

**Abstract:**

 In the paragraph about the methods, the other analyses that were conducted, apart from the proximate and ultimate analyses, should be mentioned.

**Introduction:**

 In the last paragraph, the scope of your work should have more details:

- Indicate the reasons to analyse specifically the faecal sludge from the province of Tamil Nadu;
- Give a definition of “biosolids” (too general term) and indicate the cities from which the biosolids were analysed.

 

**Methods**

 The methods section should include a section to describe the statistical analysis work performed in this work (number of samples, number or replicates per analysis, calculation method of the uncertainty bars).

*Subsection “Faecal sludge sampling”*

 In the first paragraph:

- The reasons to sample the sludge in the indicated months should be justified;
- The information about sampling in Madurai is missing.

The method to determine the total solids content, described in the last paragraph, does not correspond to the method that is commonly employed for the characterisation of faecal sludge (the method derived from the standards of wastewater analysis). The reasons to follow this method (and not the method typically practiced) should be provided.

*Subsection “Biosolids sampling”*

 The periods of the year, during which the biosolids were sampled, should be indicated (in a similar way as this has been done in the case of the faecal sludge samples).

 It is not necessary to repeat the explanation of the total solids content method, which has been already described in the previous sub-section. In order to avoid repetitions and lead to a more fluent reading, the method should be only mentioned and a cross-reference to the previous sub-section should be made for the description of the procedure.

**Results and discussion**

*Subsection “Description of FS sources”*

 The first paragraph should be placed in the Methods section.

 In the last paragraph:

- The statement “The majority of the samples collected for both cities were from residential sites” should indicate if the residential sites encompass both single and multi-units, and the percentage that they represent;
- It should be specified if the findings about the distribution across categories of the sampled sources apply for both Coimbatore and Tiruppur.  

 

*Subsection “Total solids content of FS sources”*

 At the end of the first paragraph, it is stated that only 36% of the sources in Coimbatore and 11% of the sources in Tiruppur received greywater in addition to blackwater. These values lack of significance without setting a comparison basis and therefore the typical practices in India with respect to the disposal of greywater in the toilets should be indicated.

 In the last paragraph:

- it would be interesting to compare how the samples are distributed as a function of the total solids content between Coimbatore and Tiruppur;
- The geographical location related to the statement “This study did not examine the effect of different seasons on the TS content” should be mentioned (i.e. Coimbatore).

In Figure 2, I suggest to change the y-label to “% of the total samples” (instead “total samples”).

*Subsection “Elemental composition and calorific content of FS sources”*

 In the first paragraph:

- The first two sentences should be placed in the “Methods” section;
- In the case of faecal sludge from Tiruppur, the number of samples taken per source category should be provided (first paragraph);
- The rationale behind the selection of a different number of replicates between the two cities (N = 2 for Coimbatore; N = 11 for Tiruppur) should be explained.

In the second paragraph:

- The higher ash content of the sludges from this study, compared to fresh faeces, is attributed to environmental factors such as the construction practices of the onsite sanitation systems. However, this explanation is incomplete as other factors could be also involved. For example, faecal sludge is usually biodegraded during its storage and, in consequence, its ash content tends to increase.
- The supposed correlation of the fixed carbon and oxygen content with the HHV is incorrect. A higher fixed carbon is usually associated to a higher caloric value, as demonstrated by Demirbas (2008) ^1^, while a higher oxygen content tends to lower the calorific value (because the degree of oxidation of carbon is lowered with the presence of oxygen atoms in the molecular structure of a fuel, leading to a lower energy released during its oxidation). An interesting measure of the calorific value is through its O/C and H/C. Usually, when the O/C increases and the H/C decreases, the calorific value of the material decreases (and viceversa).

In the last paragraph, it is stated that the public toilets could be expected to have a higher urination frequency than the other units. This statement should be supported with explanations or references.

 Figure 4 should include the uncertainty bars for the LHV.

Is the work clearly and accurately presented and does it cite the current literature?

Yes

If applicable, is the statistical analysis and its interpretation appropriate?

Partly

Are all the source data underlying the results available to ensure full reproducibility?

Yes

Is the study design appropriate and is the work technically sound?

Yes

Are the conclusions drawn adequately supported by the results?

Yes

Are sufficient details of methods and analysis provided to allow replication by others?

Yes

Reviewer Expertise:

Thermal process (drying, pyrolysis, gasification, combustion, biochar); faecal sludge characterization (rheology, stickiness, physiochemical properties)

I confirm that I have read this submission and believe that I have an appropriate level of expertise to confirm that it is of an acceptable scientific standard.

## References

[1] : Relationships Proximate Analysis Results and Higher Heating Values of Lignites. *Energy Sources, Part A: Recovery, Utilization, and Environmental Effects*.2008;30(20) : 10.1080/10916460701462846 1876-1883 10.1080/10916460701462846

# Response

**Published:** 2020-01-16

**Authors:** Sonia Grego

**Affiliations:** Duke University, USA

**We thank Dr. Septien Stringel for his thorough review of the manuscript. We agree with the suggestions he proposed and have implemented them in version 2 of the manuscript, which we believe is much improved.**

**A point by point response to comments follows, in bold font.**

**Abstract:**

 In the paragraph about the methods, the other analyses that were conducted, apart from the proximate and ultimate analyses, should be mentioned.

**Spelled out that TS and heavy metal ash analysis were conducted.**

**Introduction:**

 In the last paragraph, the scope of your work should have more details:

- Indicate the reasons to analyse specifically the faecal sludge from the province of Tamil Nadu;

**Added**

- Give a definition of “biosolids” (too general term) and indicate the cities from which the biosolids were analysed.

 

**Added definition of biosolid as treated sewage sludge**

**Methods**

 The methods section should include a section to describe the statistical analysis work performed in this work (number of samples, number or replicates per analysis, calculation method of the uncertainty bars).

**A section on statistical analysis was added**

*Subsection “Faecal sludge sampling”*

 In the first paragraph:

- The reasons to sample the sludge in the indicated months should be justified;

**The reason was logistical constraint of the project and this explanation was added to the text.**

  

- The information about sampling in Madurai is missing.

**No fecal sludge was sampled in Madurai, only biosolids.**

 The method to determine the total solids content, described in the last paragraph, does not correspond to the method that is commonly employed for the characterisation of faecal sludge (the method derived from the standards of wastewater analysis). The reasons to follow this method (and not the method typically practiced) should be provided.

**Indeed this rationale was missing from the original submission. We added the explanation which is related to equipment limitation.**

*Subsection “Biosolids sampling”*

 The periods of the year, during which the biosolids were sampled, should be indicated (in a similar way as this has been done in the case of the faecal sludge samples).

**Added**

 It is not necessary to repeat the explanation of the total solids content method, which has been already described in the previous sub-section. In order to avoid repetitions and lead to a more fluent reading, the method should be only mentioned and a cross-reference to the previous sub-section should be made for the description of the procedure.

**Done. Removed paragraph and added reference to method used before.**

**Results and discussion**

*Subsection “Description of FS sources”*

 The first paragraph should be placed in the Methods section.

**The information from the first paragraph was placed in the Method section. A sentence summarizing the number of truck sampled from the study is left at the beginning of the result section to orient the reader.**

 In the last paragraph:

- The statement “The majority of the samples collected for both cities were from residential sites” should indicate if the residential sites encompass both single and multi-units, and the percentage that they represent;

**Percentage were added for residential sites comprising of both single and multi-family units.**

- It should be specified if the findings about the distribution across categories of the sampled sources apply for both Coimbatore and Tiruppur.  

**Values for the categories were added to specify values for both Tiruppur and Coimbatore.**

*Subsection “Total solids content of FS sources”*

 At the end of the first paragraph, it is stated that only 36% of the sources in Coimbatore and 11% of the sources in Tiruppur received greywater in addition to blackwater. These values lack of significance without setting a comparison basis and therefore the typical practices in India with respect to the disposal of greywater in the toilets should be indicated.

**Added a reference to a government report that describes greywater disposal practices (page 3). Added text to the manuscript  “In India, wastewater disposal practices include only the toilet effluent to be plumbed to the septic tank while greywater is discharged to soak pit or other drain (**Ministry of Urban Development 2011 **).”**

 In the last paragraph:

- it would be interesting to compare how the samples are distributed as a function of the total solids content between Coimbatore and Tiruppur;

**Added**

- The geographical location related to the statement “This study did not examine the effect of different seasons on the TS content” should be mentioned (i.e. Coimbatore).

**Added**

 In Figure 2, I suggest to change the y-label to “% of the total samples” (instead “total samples”).

**Done and graphs was changed accordingly.**

*Subsection “Elemental composition and calorific content of FS sources”*

 In the first paragraph:

- The first two sentences should be placed in the “Methods” section;

**The first sentence was moved. **

- In the case of faecal sludge from Tiruppur, the number of samples taken per source category should be provided (first paragraph);

**A sentence with the breakdown data was added.**

- The rationale behind the selection of a different number of replicates between the two cities (N = 2 for Coimbatore; N = 11 for Tiruppur) should be explained.

**“A higher number of specimen were analyzed in Tiruppur than in Coimbatore for pragmatic reasons related to an opportunity of piloting novel decentralized FS thermal treatment in that city.” This explanation was added to the manuscript**

 In the second paragraph:

- The higher ash content of the sludges from this study, compared to fresh faeces, is attributed to environmental factors such as the construction practices of the onsite sanitation systems. However, this explanation is incomplete as other factors could be also involved. For example, faecal sludge is usually biodegraded during its storage and, in consequence, its ash content tends to increase.

**We agree and the additional hypothesis was added.**

- The supposed correlation of the fixed carbon and oxygen content with the HHV is incorrect. A higher fixed carbon is usually associated to a higher caloric value, as demonstrated by Demirbas (2008) ^1^, while a higher oxygen content tends to lower the calorific value (because the degree of oxidation of carbon is lowered with the presence of oxygen atoms in the molecular structure of a fuel, leading to a lower energy released during its oxidation). An interesting measure of the calorific value is through its O/C and H/C. Usually, when the O/C increases and the H/C decreases, the calorific value of the material decreases (and viceversa).

**We agree with the reviewer that the statement was incorrect by error.  HHV increases with FC and decreases VM and ash. We have revised accordingly**.

 In the last paragraph, it is stated that the public toilets could be expected to have a higher urination frequency than the other units. This statement should be supported with explanations or references.

**We are removing this statement because the nature of sanitation practices depends on contextual factors that were not tracked in this study and we are unable to support the statement.**

 Figure 4 should include the uncertainty bars for the LHV.

**Error bars were added to figure 4**
